# SUPPORTING ARAB AMERICANS FAMILIES LIVING WITH DEMENTIA: TESTING A CULTURALLY ADAPTED INTERVENTION

**DOI:** 10.1093/geroni/igae098.0155

**Published:** 2024-12-31

**Authors:** Kristine Ajrouch, Fatmeh Barada, Mary Janevic, Toni Antonucci

**Affiliations:** University of Michigan, Ann Arbor, Michigan, United States; University of Michigan, Ann Arbor, Michigan, United States; University of Michigan, Ann Arbor, Michigan, United States; University of Michigan, Ann Arbor, Michigan, United States

## Abstract

Culturally adapted interventions are needed to address unique challenges faced by minoritized groups who care for people living with Alzheimer’s disease and related dementias (ADRD). Among Arab Americans, limited access to support services due to language and cultural barriers often results in a belief that family alone is the only option during times of need. We test 1) whether participation in an adapted ADRD care intervention is associated with positive outcomes for Arab American care partners (N=58) and 2) evaluate program satisfaction. We present a single group design using paired t-tests that demonstrate reduced care burden and increased care satisfaction from pre- to post-intervention. A measure of family conflict trended downward after program participation. Following the program, each participant completed a short evaluation survey sent vias email where they (N=43) rated their experience with the program on multiple dimensions: usefulness of the program, materials, language of delivery, and Alzheimer’s Association resources. Respondents were also asked to explain their ratings, which were coded into four themes: Resources, Information regarding diseases, caregiving, and self-care. Data were analyzed descriptively and showed strong positive reactions to the program as well as areas for improvement. Findings provide preliminary data to illustrate that culturally adapted ADRD care intervention programs hold promise to help family members caring for a person living with dementia even when such programs not generally accepted by cultural norms.

